# Collectanea Medicia: Consisting of Anecdotes, Facts, Extracts, Illustrations, &c

**Published:** 1822-08

**Authors:** 


### COLLECTANEA MEDICA:

CONSISTING OF

ANECDOTES, FACTS, EXTRACTS, ILLUSTRATIONS, &c.

*Relating to the History or the Art of Medicine, and the Collateral Sciences.*

Floriferis, ut apes, in saltibus omnia libant,

Omnia nos, itidem, depascimur aurea dicta.

---

### ART. I.—*On the Effects of an Overdose of Digitalis.* By THOMAS M. FOGO, M.D. late Surgeon, Royal Artillery.

MR. K——, a respectable individual, and school-master of a parish in East Lothian, was labouring under a very severe attack of asthma when I first saw him, which succeeded a slight pneumonic affection, and for which he had been bled, &c. by my friend, Dr. Lewins, of Haddington. When I saw him, he complained of the frequent returns of difficulty of breathing during the day, particularly upon placing himself in a recumbent posture; and of their violence and quick succession during night being so great as to preclude all attempts to sleep, or indeed to remain in bed. Antispasmodics were inefficacious; opiates I could not continue, because they induced that disposition to unpleasant visions peculiar to particular habits; hyosciamus, in large doses, failed of giving even temporary quiet, though variously combined, as with ether, camphor, the camphorated tincture of opium, and others; emetics had the effect of diminishing the violence of the nocturnal paroxysms, and favouring a quieter night, though far from being followed by refreshing sleep; but such remission I could not venture every night to procure, because the patient was well advanced in years, was much worn out by the want of repose and incapability of taking sustenance, and was becoming œdematous in the lower extremities, the consequence of debility.

His son, a surgeon, conceiving digitalis might be tried, brought a phial of the tincture when he came to visit his father: as I had, however, before given it a fair trial, and the œdema appeared not to arise from any hydropic disposition, the phial was laid aside without further consideration.

On the morning of the 22d August, according to the patient's account, after having passed a most uncomfortable night, the greater part of which he was walking about in great agony, he flew to the place where several bottles were standing, and, wishing for relief, without hesitation swallowed the contents of the bottle with digitalis, which, by after measurement, we found to contain exactly an ounce, and which his son had procured from an apothecary for that quantity.* This took place about seven o'clock in the morning, as I afterwards understood.

I called at his house about eleven o'clock, when I was told he was asleep; but heard nothing of his having prescribed for himself, and therefore did not repeat my visit till past one, when I found him awake and quite easy, being then in bed. In the course of my inquiries, he told me he had to thank himself for procuring his composed state, which my medicines had failed to do; and detailed the draught which he had taken early in the morning. I then found he had fallen asleep almost immediately after taking the dose, slept soundly till half-past ten, when he vomited a good deal, and likewise had an evacuation *per anum*, and very composedly afterwards resumed his bed and the enjoyment of repose. Upon being satisfied of what he had done, I lost no time in administering a powerful emetic, which operated speedily, and completely cleansed the stomach of its contents: at this time his pulse was 80, and perfectly regular. I saw him again at four o'clock, when he continued in the same tranquil state, and between my visits had enjoyed some sleep: the pulse was as before. I repeated my visits at six o'clock, when I found him quite easy, free from vertigo and nausea; nor had he had any alvine evacuation since I saw him last: his pulse, in frequency and strength, was much the same, though every two or three minutes there was a slight intermission. I now began to dread the effects of the medicine on the action of the heart, and ordered some strong punch to be made, which was given in divided doses. About half-past eight o'clock I saw him again, when the only change observable was in the pulse, which now intermitted more frequently, and the intermissions were longer, though seldom more than one each minute.

To prevent the sedative effects of the digitalis, I considered the only line of practice to be pursued; though, I confess, I had no great hopes of success in my endeavours; but I was determined to be at hand, and remained by the patient's bedside the whole night. With this view I began, about nine o'clock, to give him repeated doses of the carbonas ammoniæ, of which I gave him grs. x. in the form of a bolus; but afterwards changed it for the aqua ammoniæ, of which I gave gtts. xx. alternated with ʒiss. of the ether nitrosus, every hour. After he had taken two doses of medicine, I began to mark particularly the variations

* Dr. Lewins was good enough to make inquiry at the shop from whence the medicine was obtained, and he was assured that the tincture was in a fresh state, and made the season before, from leaves gathered at the proper time.

2

of the pulse, which had now changed much, in the frequency of the intermissions, in their length, and consequently in the number of pulsations during a minute.

As I had dispatched an express for his son, when I began giving the stimulating medicines, his arrival before midnight was satisfactory, for he enabled me to pay all proper attention to his father.

The medicines were administered regularly every hour, and in the intervals the patient slept, upon the whole, tolerably composed; and, when he was awoke to take again the draught, always assured us he felt no uneasiness of any kind. The state of the pulse, as noted every half hour, will show how variable it was, even in the course of a few minutes, to eight or ten beats: we therefore preferred stated observations, from which it will be remarked that the sinking of the pulse was by no means progressive, as would probably have been the case if the medicine had been left to itself; and which variation at times led me to encourage hopes that we might be successful in our counteracting plan, remembering, as I did, the case of a young lady, as noticed by the late distinguished Dr. Gregory in his Lectures, whose pulse suddenly was diminished to twenty-eight pulsations in the minute, from the accumulation of repeated doses of digitalis, and who nevertheless recovered by the free exhibition of brandy.

State of the pulse, as noted each half hour:

| | | | | | |
|---|---|---|---|---|---|
| At 11 | o'clock | 58 | half-past | 56 | |
| — 12 | — | 52 | — | 46 | |
| At 1 | A.M. | 42 | — | 55 | |
| — 2 | — | 43 | — | 66 | |
| — 3 | — | 42 | — | 50 | |
| — 4 | — | 45 | — | 41 | |
| — 5 | — | 36 | — | 41 | |
| — 6 | — | 59 | — | 47 | |
| — 7 | — | 47 | — | 48 | At 7¼ — 72.* |
| — 8 | — | 42 | — | 48 | |
| — 9 | — | 46 | — | 53 | |
| — 10 | — | 69 | — | 74 | Took tea. |
| — 11 | — | 73 | no intermission | — | |
| — 12 | — | 75 | — | — | |

By twelve o'clock the following day, I considered we had completely counteracted the sedative effects of the digitalis, and therefore discontinued particularly remarking the pulse, though from that time it continued pretty steady; and contented myself with merely prescribing repeated doses of the mixtura camphorata; which, however, I intermitted in the course of twenty-four hours, every danger to be apprehended being by that time removed. Since that period to the present time there never has been the least untoward symptom; and, besides being as it were dragged from the grave, he enjoys wonderfully good health, and is able to go about his usual avocations, without any asthmatical affection whatever.

* The pulse being at 72 at a quarter-past seven, is to be accounted for from his exerting himself in bed, which in fifteen minutes afterwards fell to 48.

It is a desideratum how far we may be justified in giving digitalis in large doses in cases of asthma, and to what extent it can be given with safety? From a case which has come to my knowledge, which happened in this country some years ago, where a patient took a teaspoonful by mistake, in place of paregoric, without inducing any very alarming symptoms, and from the instance I have now related, it will perhaps be found that this drug may be given in quantities almost beyond human credibility, and with the happiest effect; while, from small repeated doses, more alarming symptoms will be produced than from many times the quantity given at once. Further observation can only resolve this point.

Fortunate as has been the issue of this case, yet I cannot but regret that I did not see the individual when I first called in the morning; for I might have been enabled to set at rest the long-disputed point, whether it first acts as a stimulant? When I did see him, the pulse was the same as it had been for many days before; and, if it had raised the pulse in the first instance, it had returned to its wonted state by that time. Allowing, however, that I had not a fair opportunity of judging, yet I think the facts militate against any such stimulating properties of the foxglove, because, in my idea, the stimulating ought to have been more quickly succeeded by the sedative effects: of the latter there is no doubt, but the former is still *sub judice.*—(*Edinburgh Med. Journal,* No. lxxii.)

ART. II.—*Case of Purpura Hæmorrhagica.* By GEO. JOHNSTON, M.D. Surgeon, Extraordinary Member of the Royal Medical Society of Edinburgh.

SUNDAY, February 3d, 1822, 10 A.M. Mary Gill, aged eighteen, married, of a stout and robust form, complains of intense pain in the head and small of the back, attended with great oppression in the chest, and the feeling of burning heat over the whole body. The skin is red, something like that of a person labouring under scarlet fever; and the whole surface, with the exception of the face and arms, is closely covered with innumerable petechiæ, some of a bright red, others of a purple colour, and varying in magnitude from a mere point to a silver penny. Her arms are quite free of them, but sprinkled over with a considerable number of small, elevated, circular spots, resembling, in every respect, those produced by the sting of the nettle. The conjunctiva of both eyes is in a state of excessive ecchymosis, so that no part of the white can be seen. The pupils are contracted, but no complaint is made of the light. She has had no delirium. The tongue is covered, partly with a dark brown, partly with a white, fur; has some cough, and the sputa are bloody. The lips are parched and rather livid, and blood oozes from the nostrils. Respiration is very rapid, and performed with difficulty; the pulse is so quick and weak that it cannot be numbered. No passage in her bowels has taken place since the commencement of the disease; and the urine is scanty, and deeply tinged with blood. Catamenia ceased fourteen days ago.

On the evening of Sunday last, she was seized with head-ach and cold shivering, succeeded by the ordinary symptoms of fever,—increased heat of skin, anorexia, and pain of the back and lower extremities. She continued in this state, yet not so unwell as to be entirely confined to bed, until Thursday, when an eruption, compared by the attendants to that of the measles, was observed to cover the whole surface, attended with an increase of all the febrile symptoms. On Saturday every symptom became aggravated; and it was now for the first time that the petechiæ and wheals were noticed. An injection was administered in the morning, which produced no effect; and in the evening she took four of the pilul. colocynth. comp., and two every six hours subsequently, but without producing any evacuation from the bowels.

Fourteen ounces of blood were immediately taken from the arm, which induced a strong tendency to syncope. On recovering from this, she felt herself much relieved: the oppression in the chest was altogether removed; the breathing rendered comparatively easy; the redness of the skin disappeared; and a little of the white of the conjunctiva became apparent. The bleeding from the arm was suppressed with difficulty.

℞. Calomelanos, gr. v.
Pulv. jalapæ, ℈j. Ft. pulvis statim sumendus.

*Half-past* 3 P.M.—Patient apparently dying. She is however sensible, though it is with great difficulty she can be roused to answer any question. Complains of a pain in the throat; and, on examination, a diffused redness covers the whole upper palate; has spit blood copiously; face and lips pale, and somewhat livid; respiration fuller, and not so quick as in the morning; pulse very weak, and exceedingly frequent; surface of the body rather cold, but not clammy. A little blood, not exceeding an ounce or two, has escaped from the orifice in the arm; the purgative has had no effect. The blood drawn in the morning has not separated into serum and crassamentum. It possesses little consistence or tenacity, but there are traces of coagulable lymph diffused through it. Every attention had been paid to obviate the circumstances which might prevent the formation of a buffy coat.

At the request of a gentleman interested in the patient, Dr. Robertson now saw her. He directed an injection of a decoction of senna with sulphate of soda, and hot bottles to be applied to the feet; but, before the former could be prepared, the approach of death became so evident to the relatives, that it was not exhibited. She expired a little before six.

I opened the body twenty hours after death, in the presence of Dr. Robertson. About a pint of bloody serum was found in each cavity of the pleura; the lungs were perfectly sound, and not adherent any where to the costal pleura. There was a blush of redness on the anterior surface of the pericardium, which contained between two and three ounces of serum. The heart was paler and smaller than usual, and was lacerated by slight exertions of the fingers. It was spotted with petechiæ, some red, others purple. They were larger than those of the skin, and most conspicuous about the middle of the left ventricle, and at the roots of the large vessels. The left ventricle was empty; the right was distended by a quantity of uncoagulated blood. Nothing could be more healthy than the exterior appearance of the abdominal viscera. The mesentery was thickly spotted with petechiæ, (particularly near its connexion with the intestines,) some of which were at least half an inch in diameter. The peritoneum was also spotted, particularly in the neighbourhood of the spleen, where it was indeed entirely purple. The inferior surface of the liver, and the internal surface of the stomach, were also covered with maculæ. The kidneys appeared larger than usual, and the medullary and cineritious portions more distinctly defined than in the healthy organ. The urinary bladder was empty; the uterus unimpregnated. On tracing the intestinal canal, no feces could be discovered throughout its whole extent; no emaciation had taken place; and the cellular membrane was loaded with fat. We regretted much that we could not examine the brain. The prejudices against opening dead bodies are here so strong, and consent in this instance was so reluctantly given, that it could not with propriety be done.—(*Edinburgh Med. Jour.* No. lxii.)

---

ART. III.—*Case of Rupture of the Larynx Trachea by external Violence, inducing Emphysema.* By LUCIUS O'BRIEN, M.D.

H. G. was brought into the Royal Infirmary of Edinburgh, on the 23d July, 1818, about half-past five o'clock P.M. in the following state:

Face very tumid, especially on the right side; right eyelids so distended as nearly to close the eye; lips livid; breathing very laborious and stridulous, most easily performed in the sitting posture. She complained of thirst, and repeatedly asked for water, which, however, she was unable to swallow.

On being examined more particularly, the skin in general was cold, and, on the thorax, neck, face, and superior extremities, distinctly emphysematous; and the pulse was scarcely perceptible. When questioned as to where she was hurt, she pointed to her throat, and said she had been kicked under the jaw and in the belly, and much bruised on the right side. When pressure was made on the right side of the thorax, about the middle of the ribs, she complained of pain, but no crepitus could be distinguished. Pressure on the abdomen caused considerable pain, but no mark of injury could be perceived. She could hardly bear any examination of her neck, which was very tumid; and on the lower anterior part of it was a bluish mark. She had been bled before admission, and some leech-bites were observed on her breast.

Some scarifications were made about the lower part of the neck, and a small quantity of warm wine and water were given. After some time, the emphysema seemed somewhat less, and the pulse became more distinct, but was intermitting. About half-past nine a full opiate was administered, and some toddy directed to be given at intervals during the night.

*24th July, half-past eight* A.M.—Seems much in the same state as last night, and is reported to have had vomiting. After half-past eleven o'clock became suddenly more faint, and her breathing more difficult; and very shortly after expired.

*Dissection*, 25th July, in presence of Dr. Duncan, junior, and Messrs. Law and Gillespie, surgeons to the Infirmary:

There was distinct ecchymosis of the soft parts on the lower anterior part of the neck, extending down to the muscles over the trachea; and the cellular substance was every where distended with air. On the soft parts being removed, a rupture was found through the whole depth of the thyroid and cricoid cartilages, on the left of their mesial plane, and then through the right side of the first ring of the trachea.

A transverse incision was made through the soft parts above the os hyoides; and the pharynx, œsophagus, larynx, and trachea, raised up from the spine. On slitting up the œsophagus and pharynx posteriorly, an effusion of blood was found under the mucous membrane of the left side of the pharynx, of an irregular figure, and occupying, perhaps, the extent of a square inch. It extended to the inside of the epiglottis, between it and the arytenoid cartilage for a quarter of an inch. Here the membrane was much tumefied, and projected into the passage, so that it must have obstructed respiration very considerably. Another effusion, but much smaller, was found on the middle of the right posterior edge of the thyroid cartilage. The larynx and trachea were then opened posteriorly; and the membrane lining them, and the pharynx, were more than usually vascular, and of a light-pink colour. The ruptured edges of the cartilages were rather tumefied, and slightly inverted. Most of the cellular substance surrounding the larynx, pharynx, and trachea, was emphysematous.

The right lung was found collapsed, but no laceration of its surface, or fracture of the ribs, could be detected; and the lung was capable of distention by being inflated. Through its substance were a number of small hard tubercles; but it appeared healthy in other respects, there being no adhesion between it and the pleura costalis, nor any fluid in that cavity of the thorax.

The left lung was extensively diseased, its substance being full of tubercles and vomicæ, and it adhered by almost all its thoracic surface to the pleura costalis.

The anterior mediastinum was completely emphysematous. On opening the heart, the blood was found not coagulated, and quite frothy from the admixture of air; and that in the aorta and vena cava abdominalis was in the same state.

In the head, the only morbid appearance was some turgescence of the vessels in the ventricles; and the arteries were distended with very dark fluid blood, like that in the veins.—*Edinburgh Med. J.* No. lxxii.

